# Letter from Dr. N. S. Jenkins, Dresden, Germany

**Published:** 1903-07

**Authors:** N. S. Jenkins

**Affiliations:** Dresden


### Foreign Correspondence.

### LETTER FROM DR. N. S. JENKINS, DRESDEN, GERMANY.

To the Editor:

Sir,—In the May number of the International Dental Journal, page 365, appears a letter from Dr. Rollins which requires correction.

The translation of Dr. Bruck's article which appeared in *Items of Interest* in May, 1902,—not 1892, as Dr. Rollins says by a slip of the pen,—was erroneously attributed to me. It was, however, made by Dr. Charles W. Jenkins, of Zurich, and, in the publication of the article in book form, he is acknowledged as the translator. I have attentively read both the original and the translation, and see no evidence that the translator has, in any particular, taken the unjustifiable course of either adding to or detracting from the original text or offering any opinion upon its accuracy.

There is a great resemblance in the history of all important discoveries. Different minds work upon the same problem, often for many years. Here and there a gleam of truth is seen, and some investigators are almost upon the secret when they are turned aside by uncontrollable circumstances, until at last it is given to one to combine the fruits of many laborers and to obtain the desired result. In the profound absorption with which I have carried on my investigations for so many years I have never paused to inquire as to the priority of any idea I have adopted, being grateful with a very deep gratitude to men like Rollins and Land, as well as many others, who have thrown light upon the path I was pursuing; for I have frankly appropriated, wherever I found it, any suggestion which could facilitate my progress. It would be impossible to enumerate the sources from which I have received aid, ranging from the humblest mechanic to the most gifted scientist, but I have repeatedly stated in public that the accomplishment of the task I had set myself was largely due to the discoveries, the suggestions, the advice, and the sympathetic co-operation of many far abler than myself. My only merit is that, being so situated, I could carry on a long series of experiments under circumstances more favorable than fall to the lot of most men. I have been my own severest critic, and have persevered in a work which could only have been done by a dentist until I have convinced myself that the result is of the highest importance.

N. S. Jenkins.

Dresden, June 3, 1903.

